# Systematic Review of Studies Reporting Positive Surgical Margins After Bladder Neck Sparing Radical Prostatectomy

**DOI:** 10.1007/s11934-017-0745-0

**Published:** 2017-11-07

**Authors:** Mariangela Bellangino, Clare Verrill, Tom Leslie, Richard W. Bell, Freddie C. Hamdy, Alastair D. Lamb

**Affiliations:** 1 Department of Urology, Sant’Andrea Hospital, Sapienza University of Rome, Rome, Italy; 2 Department of Urology, Churchill Hospital, Oxford University Hospitals NHS Foundation Trust, Oxford, UK; 3 Department of Pathology, John Radcliffe Hospital, Oxford University Hospitals NHS Foundation Trust, Oxford, UK; 4 Nuffield Department of Surgical Sciences, University of Oxford, Oxford, UK

**Keywords:** Bladder neck preservation, Bladder neck sparing, Positive surgical margin, Prostatectomy, Oncological outcome, Biochemical relapse

## Abstract

**Purpose of Review:**

Bladder neck preservation (BNP) during radical prostatectomy (RP) has been proposed as a method to improve early recovery of urinary continence after radical prostatectomy. However, there is concern over a possible increase in the risk of positive surgical margins and prostate cancer recurrence rate. A recent systematic review and meta-analysis reported improved early recovery and overall long-term urinary continence without compromising oncologic control. The aim of our study was to perform a critical review of the literature to assess the impact on bladder neck and base margins after bladder neck sparing radical prostatectomy.

**Evidence Acquisition:**

We carried out a systematic review of the literature using Pubmed, Scopus and Cochrane library databases in May 2017 using medical subject headings and free-text protocol according to PRISMA guidelines. We used the following search terms: bladder neck preservation, prostate cancer, radical prostatectomy and surgical margins. Studies focusing on positive surgical margins (PSM) in bladder neck sparing RP pertinent to the objective of this review were included.

**Evidence Synthesis:**

Overall, we found 15 relevant studies reporting overall and site-specific positive surgical margins rate after bladder neck sparing radical prostatectomy. This included two RCTs, seven prospective comparative studies, two retrospective comparative studies and four case series. All studies were published between 1993 and 2015 with sample sizes ranging between 50 and 1067. Surgical approaches included open, laparoscopic and robot-assisted radical prostatectomy. The overall and base-specific PSM rates ranged between 7–36% and 0–16.3%, respectively. Mean base PSM was 4.9% in those patients where bladder neck sparing was performed, but only 1.85% in those without sparing.

**Summary:**

Bladder neck preservation during radical prostatectomy may increase base-positive margins. Further studies are needed to better investigate the impact of this technique on oncological outcomes. A future paradigm could include modification of intended approach to bladder neck dissection when anterior base lesions are identified on pre-operative MRI.

## Introduction

Prostate cancer is the most frequently diagnosed non-cutaneous cancer and the second leading cause of cancer-related death in men in the western world [1]. Radical prostatectomy (RP) is the standard surgical treatment for patients with localised prostate cancer and life expectancy of more than 10 years [2]. Radical retropubic prostatectomy (RRP) has long been the most commonly used surgical approach. New surgical techniques, such as laparoscopic radical prostatectomy (LRP) and, more recently, robot-assisted radical prostatectomy (RARP), have reduced the morbidity and permitted detailed refinement of key steps in the procedure [3]. The optimal outcome for clinically localised prostate cancer is freedom from biochemical recurrence (BCR), recovery of continence and erectile function, no perioperative complications and absence of positive surgical margins—the pentafecta. These are only achieved in 62–70% of patients [, 4, 5]. Bladder neck sparing (BNS) techniques were first reported in 1992 in attempt to preserve the internal urethral sphincter for improving post RP urinary continence [6]. Since the introduction of this approach, several authors reported improved early recovery of urinary continence without compromising cancer control [7–13]. However, some studies reported little difference in improving continence and suggested an increased risk of positive surgical margins [14–16]. Positive surgical margins after RP are consistently and independently associated with higher risk of biochemical recurrence (BCR), although their impact on long-term outcomes including metastasis, castrate-resistant prostate cancer and prostate cancer-specific mortality remains controversial [17–24]. Nonetheless, a positive surgical margin is undoubtedly a source of anxiety for patients who consequently need close monitoring with close PSA surveillance and, occasionally, additional treatment such as adjuvant or salvage radiotherapy [2]. A previous systematic review and meta-analysis of 13 studies assessing bladder neck sparing (BNS) versus non-BNS techniques reported improved early and long-term recovery of urinary continence without negatively affecting oncological control. Their results were yet non-conclusive given the limitations of the studies included, and the authors suggest the need for further large, prospective, multicentre, long-term follow-up studies and RTCs to confirm them [25]. Here, we perform a critical review of the available data localising positive surgical margins after bladder neck sparing radical prostatectomy.

## Evidence Acquisition

**Fig. 1:**
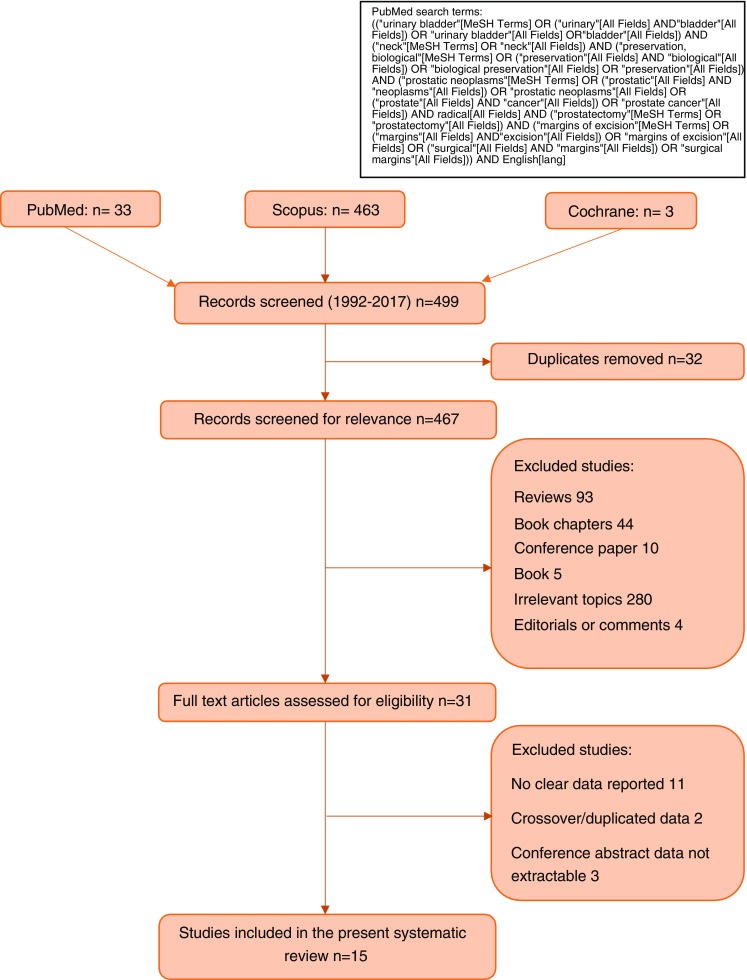
Identification and screening of studies (PRISMA)

We performed a systematic review of the literature in May 2017 using Pubmed, Scopus and Cochrane databases, including medical subject headings and free-text protocols. The search was conducted according to the Preferred Reporting Items for Systematic Reviews and Meta-Analyses criteria for systematic reviews (http://www.prisma-statement.org) and was restricted to the following terms: bladder neck preservation, prostate cancer, radical prostatectomy and surgical margins. We set the limits: gender (male), subject (medicine) and English language. Two authors (MB and ADL) independently reviewed the abstracts of the obtained studies and selected those that were related to the topic of the present review. The corresponding full-text articles were assessed by the authors. Abstracts, conference reports, duplicated data and articles on irrelevant topic or reporting no clear data for our objective were excluded, including in the final analysis only articles reporting complete data clinically relevant for this review (Fig. 1).

## Evidence Synthesis

Overall, 15 studies that reported overall and site-specific positive surgical margin rate after bladder neck sparing radical prostatectomy were considered relevant for the present review. This included two RCTs, seven prospective comparative studies, two retrospective comparative studies and four case series (three prospective and one retrospective). Included studies were published between 1993 and 2014 and sample sizes ranged between 50 and 1067. Surgical approaches included open (eight studies), laparoscopic (three studies), robot-assisted (three studies) and both open and robot-assisted radical prostatectomy (one study). The overall and base-specific PSM rates ranged between 7–36% and 0–16.3%, respectively. The rate of PSM specifically found only at the bladder neck ranged between 0 and 10%.

## Reporting on Bladder Neck Sparing Technique

Bladder neck sparing technique evolved from a better knowledge of the anatomy and physiology of the mechanisms involved in urinary continence. The bladder neck is an integral part of a larger and complex sphincter mechanism. The urethral sphincter is composed of an inner lissosphincter of smooth muscle and an outer rhabdosphincter of skeletal muscle. It extends in the form of a cylinder around the urethra from the vesical orifice to the distal part of the membranous urethra. While the outer component of skeletal muscle is most marked and thickest around the membranous urethra and becomes gradually less distinct toward the bladder, the inner component of smooth muscle is more represented at the vesical orifice and is thinner in its further course in the urethra. The rhabdosphincter is horseshoe-shaped and overlies the circular and longitudinal smooth muscles of the urethra. During periods of increased intra-abdominal pressures, it contracts and coapts to the urethra. The lissosphincter, on the other hand, maintains urethral resistance and is the primary mechanism responsible for maintaining resting and baseline continence [26]. The aim of BNP technique is to leave most of the lissosphincter mechanism intact, thus allowing preservation of its function and improving continence. Since its introduction in the early 1990s, it has been extensively adopted in open, laparoscopic and robot-assisted radical prostatectomy, with circumferential, lateral and anterior approaches described. It is performed by a careful dissection and preservation of the circular bladder neck fibres from the prostate base, thus obtaining a bladder neck circumference that approximates the urethral stump permitting direct anastomosis without the need for further bladder neck reconstruction [8–16]. Even considering the advantages of 3-dimensional ×12 magnification of the robotic system that could provide better discrimination between bladder neck fibres and prostate tissue, the absence of a tactile feedback makes bladder neck dissection one of the most challenging steps of RARP, especially for surgeons in their learning curve [27, 28]. As regards indications and contraindications for BNP procedure, there is no unanimous agreement; however, some studies have urged caution in the presence of extraprostatic extension which increases the risk of PSM [29]. Other areas for caution include previous pelvic or transurethral prostate surgery that may increase the technical difficulty [12, 30, 31].

## Reporting on Site of PSM in Radical Prostatectomy Specimen

**Fig. 2:**
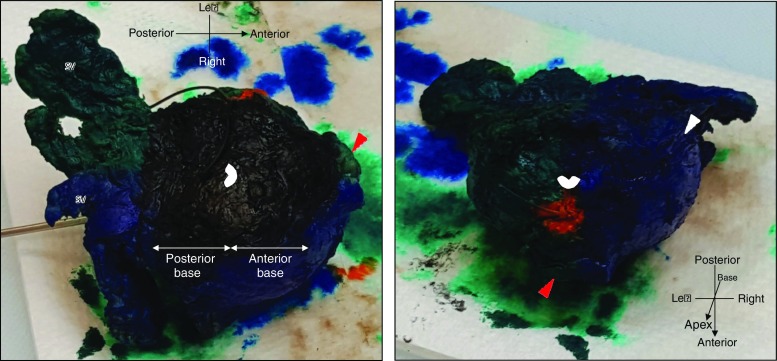
Radical prostatectomy specimen preparation. Inking performed to maintain orientation during sectioning. Blue = right, green = left, black = base. Areas of suspected capsular incision are marked by histopathology technician in orange (indicated by surgeon with suture). Urethra is marked in white. This was a non-bladder neck sparing procedure—obvious bladder apron visible on anterior base (red arrow) to avoid a positive margin from tumour identified on MRI and transperineal biopsy in an anterior location near the base. This was procedure non-nerve sparing with both neurovascular bundles visible on the specimen (white arrow)

Histopathological reports of RP specimens describe the pathological stage, cell type and Gleason grade of prostate cancer and surgical margins. Total or partial embedding of the prostate can be performed, depending on the capability of the institution to dissect and archive whole amount specimens. In order to demonstrate surgical margin status, the entire RP specimen has to be inked upon receipt in the laboratory (Fig. 2). Specimens are fixed by immersion in buffered formalin for at least 24 h, preferably before slicing. More homogeneous fixation can be provided by injecting formalin and sectioning after 24 h. Techniques vary, but in our unit, the apex and the base (bladder neck) are removed and cut into para sagittal or radial sections. The remainder of the specimen is cut in transverse, 3–4 mm sections, perpendicular to the long axis of the urethra. The resultant slices can be embedded and processed as whole mounts or after quadrant sectioning [32]. Surgical margins are considered positive only if cancer cells touch the surface of the RP specimen (tumour on ink). The location of the positive margin should be specified in the pathology report and stratified as posterior, posterolateral, lateral, anterior, apical, base or bladder neck. Although there is no evidence that the site of the positive margin affects the prognosis, it is essential information for technique development [2, 33].

There is contrasting evidence regarding the relationship between margin extent and recurrence risk [34, 35]. However, the European Urology Association guidelines currently recommend that information on multifocality and extent of margin positivity, such as the linear length of involvement in 1 mm (focal ≤ 1 mm versus non-focal > 1 mm) or number of blocks with positive margin involvement, should be given [2, 33]. For the purpose of our review, we only considered studies clearly reporting base-specific positive margins, as logic suggests that the BNP technique should affect this site compared to other locations.

## Differentiating Sites of PSM

The overall and base-specific PSM rates in the studies reviewed ranged between 7–36% and 0–16.3%, respectively. Seven studies reported the rate of PSM specifically found only at the bladder neck and it ranged between 0 and 10%. None of these studies, however, differentiated between anterior and posterior base PSM. Most of the studies included only patients with clinically localised prostate cancer [10, 11, 16, 30, 36–38], whereas five studies also included some patients with cT3 disease [12–14, 39, 40] and two did not clearly report clinical stages [9, 41]. Pre-operative assessment of clinical stage with abdominal and pelvic computerised tomography (CT) or magnetic resonance (MR) was reported in two studies [14–16], while it was employed in some cases in the studies by Gomez et al. and Bianco et al., and it was not mentioned in the remnant studies. Pre-operative MRI was not performed routinely in any study.

## Suitability of BNP

BNP radical prostatectomy was extensively performed in most studies [9, 11, 16, 36, 38–40, 42]. Exclusion criteria reported in some studies were history of previous prostatic surgery [10, 12–14, 30, 41], large mid-prostate lobe [10, 14, 30, 37] and high-risk prostate cancer (defined as PSA > 20 ng/ml or clinical T3 or clinical Gleason score > 7) [10, 13]. Selli et al. included for BNP certain patients with clinical T3a tumours after exclusion of bladder neck involvement by using transrectal ultrasonography with cystourethroscopy and biopsy [13].

## BNP and PSM

Table 1 summarises the relationship between BNP in radical prostatectomy and positive surgical margins in the studies reviewed. Katz et al. reported data on a prospective series of 235 patients who underwent LRP between 1998 and 2001. During 2000, they stopped preserving bladder neck, and they found that by 2001, the rate of PSM at bladder neck fell from 9.75 to 0% [16]. Their series, however, was consecutive rather than randomised and possible bias related to the increasing experience of the surgeon cannot be excluded. Similarly, Srougi et al. in their RCT found that bladder neck margins were positive for tumour in 6 of 70 patients, including 4 from the BNP group and 2 from the non-BNP group. Although the difference between groups in the rate of positive bladder neck margins was not statistically significant (*p* = 0.40), margins were positive at the bladder neck alone in 3 of the 31 (10%) patients from the BNP group and in none of the 39 from the non-BNP group (*p* = 0.08). This data raised ethical concerns and prompted the investigators to halt the study early [14].

**Table 1:** Systematised data

Author	Year	Sample size (BNP/non-BNP)	Design	Surgical technique	Overall PSM	bPSM		*p* (Fishers Exact)
						BNP	Non-BNP	
Gomez	1993	50	Prospective	ORP	18 (36%)	3 (6%); 0 only BN	–	n/a
Licht	1994	206 (114/83)	Prospective	ORP	–	9 (7.9%); 0 only BN	5 (6%); 0 only BN	0.61
Shelfo	1998	365	Retrospective	ORP	119 (33%)	27 (7%); 2 (0.5%) only BN	–	n/a
Freire	2009	619 (348/271)	Prospective	RARP	79 (12.8%)	5 (1.4%)	6 (2.2%)	0.45
Srougi	2001	69 (31/38)	RCT	ORP	–	4 (13%); 3 (10%) only BN	2 (5%); 0 only BN	0.24 0.047
Katz	2003	235	Prospective	LRP	62 (26.3%)	9.75%	0	n/a
Bianco	2003	555	Prospective	ORP	178 (32%)	13 (2%); 2 (0.4%) only BN	–	n/a
Selli	2004	131	Retrospective	ORP	30 (22%)	7 (5%) only BN	–	n/a
Stolzenburg	2010	240 (150/90)	Retrospective	LRP	25 (10.4%)	1 (0.7%) only BN	1 (1%) only BN	0.802
Chlosta	2012	194	Prospective	LRP	14 (7%)	0	–	n/a
Nyarangi	2012	208 (95/104)	RCT	RARP/ORP	28 (13.5%)	2 (2%) only BN	0	0.15
Friedlander	2012	1067 (791/276)	Prospective	RARP	147 (13.8%)	9 (1.1%)	7 (2.5%)	0.094
Golabek	2014	295	Retrospective	LRP	86 (29.15%)	14 (16.3%); 2 (2.3%) only BN	–	n/a
Brunocilla	2014	80 (40/40)	Prospective	ORP	13 (16%)	0	0	1
Lee	2014	599 (581/18)	Retrospective	RARP	105 (17.5%)	8 (1.4%)	0	0.61
					Mean bPSM	4.89%	1.86%	
					Median bPSM	2%	1%	

Other authors, however, did not confirm these findings and reported that BNP technique did not negatively correlate with PSM. Gomez et al. performed a prospective study on 50 patients undergoing BNP open radical prostatectomy. In their series, the overall rate of PSM was 36%, but the bladder neck was involved only in 3 patients (6%) and all of these had PSM elsewhere indicating that these might simply have been difficult cases with widespread extensive disease. The high rate of positive margins in this study is almost certainly related to the high rate of T3 disease (25%) [39]. Similar findings were described by Licht et al. in a prospective cohort of 206 patients. Base PSM rate was 7.9% in the BNP group, but in no patients was it the only positive site. Bladder neck involvement was also associated with higher stage, more than 50% likelihood of seminal vesical involvement and a higher incidence of lymph node metastases [30]. Moreover, in their retrospective series of 365 patients treated with RRP and BNP, Shelfo et al. reported an overall and base PSM rates of 33 and 7%, respectively. The bladder neck was the only site involved in two (0.5%) of the cases [11]. A larger prospective study of 555 men undergoing RRP with BNP technique was performed by Bianco et al. and confirmed those findings. They reported a total PSM rate of 32%; however, PSM at bladder neck were found in 13 patients (2%) and it was the only location in only two patients (0.4%) [36]. In a prospective study by Freire et al. on 619 men undergoing RARP, the total and base PSM recorded were 12.8 and 1.4% in BNP group and 2.2% in non-BNP group, respectively [42]. A large prospective study by Friedlander et al. on 1067 patients reported the same overall PSM in BNP and non-BNP patients (13.8%), base-positive margins being 1.1 and 2.5%, respectively [9]. A prospective, randomised, single-blind trial was performed by the group of Nyarangi et al. in 208 patients undergoing RARP with or without BNP. They found no evidence of a difference in surgical margin status between the control and the bladder neck preservation group (12.5 versus 14.7%, *p* = 0.65). Only 2% of men presented an isolated base PSM [41]. Chlosta et al., in a prospective series of 194 patients undergoing BNP LRP, assessed a total PSM of 7%, none of them was located at the bladder neck [37].Similarly, Brunocilla et al. described an overall PSM rate of 16% and no PSM at the prostate base both in men underwent BNP and non-BNP RRP [10]. Golabek et al., in a retrospective study of 295 men undergoing BNP laparoscopic radical prostatectomy, found a total PSM rate of 29.15%. The distribution of PSM for pT2, pT3 and pT4a was 15.3 (27/176), 49.1 (58/118) and 100% (1/1), respectively. Overall, 20.0% had an isolated PSM and 13.7% had multiple positive sites. The bladder neck was a positive margin in 14 cases (16.3%), and in 12 out of those (85.7%), it was in combination with a PSM at one or two other sites [11].The relatively high incidence of PSM in this study could be attributed to a large number of extracapsular tumours (40.3%). Furthermore, as BNP was performed consistently, patients with median lobe hypertrophy or high-risk features were not excluded. Selli et al. reported an overall PSM rate of 22% in 131 men undergoing RRP with BNP technique. In this series, only seven cases (5%) were positive exclusively at the bladder neck and this subgroup included patients with more aggressive pT3a disease, two of whom also had lymph node involvement, two received 3 months of neoadjuvant androgen deprivation therapy and five presented a Gleason score > 7 [13].

The incidence of positive surgical margins described in literature is equivalent among open, laparoscopic and robotic approaches. Tewari et al. reported an overall PSM rates of 24.2% for ORP, 20.4% for LRP and 16.2% for robot-assisted laparoscopic prostatectomy (RALP; no statistical evidence of a difference). Furthermore, pT2 PSM rates were 16.6% in ORP, 13.0% in LRP and 10.7% in RALP, whereas pT3 PSM rates were 42.6% in ORP, 39.7% in LRP and 37.2% in RALP [43]. Similarly, Novara et al. reported a 15% mean rate of PSMs in RARP series with a stage-specific rate of 9% for pT2 (4–23%), 37% for pT3 (29–50%) and 50% for pT4 (40–75%), supporting the evidence that a higher stage confers a higher risk of positive surgical margins. The prevalence of PSM stratified by location was as follows: apex 5% (1–7%), anterior 0.6% (0.2–2%), bladder neck 1.6% (1–2%) and posterolateral 2.6% (2–21%). Multifocal PSM was detected in 2.2% (2–9%) of the cases [44]. As expected, most of the studies included here report a higher risk of positive surgical margins for tumours of higher grade and stage and higher PSA values [9, 11, 12, 16, 30, 36, 39].

Other clinical factors that may increase the risk of PSM include elevated BMI, large prostate, previous prostatic or abdominal surgery [31, 45, 46]. These factors are still the object of investigation in the current literature, while the surgeon experience has been more clearly linked to improved outcomes. The incidence of PSM is relatively high at the beginning of the learning curve, but it tends to reach a plateau with increasing experience. The number of procedures estimated to reduce the positive margin rate to a minimum is reported in literature with a range of 200–250 cases in laparoscopic series. As regards robotic surgery, a single-surgeon study by Thompson et al. [47] reported a plateau after 100–200 RARP in pT2 disease and after 200–300 cases in pT3 disease, and the randomised controlled trial of open versus robotic surgery suggested a plateau between 100 and 200 cases [48, 49]. However, a multicentre review of 3794 patients described a learning curve with a plateau for PSM in pT3 that was only reached after 1000 cases [50–52].

## PSM and Biochemical Relapse (BCR)

We attempted to address the association between PSM and BCR. Positive surgical margins in RP have been associated with an increased risk of PSA recurrence in several studies. [21–24, 53, 54]. Given the relatively short-term follow-up, most studies evaluating the effect of PSM on treatment efficacy reported BCR as an early end point. If untreated, BCR could anticipate clinical progression; however, the variable natural history of prostate cancer limits BCR use as a surrogate for metastatic progression and mortality. Furthermore, while BCR in the setting of PSM may suggest local recurrence, distant relapse cannot be excluded, especially when additional high-risk features, such as extensive extraprostatic extension or seminal vesicle involvement, are present. Thus, it is extremely difficult to predict the precise influence of PSM on the natural course of the disease in individual patients [33, 55]. Alkhateeb et al. reported a BCR-free survival of 93.8 and 79.9% in patients with negative and positive surgical margins, respectively [56]. However, studies directly comparing the effect of a PSM to metastatic-free survival and mortality are less conclusive, mainly due to a wide range of time to mortality or the presence of other risk modifiers.

Biochemical recurrence-free survival was reported by only four of the studies included in the present review, two of these comparing BNP group versus non-BNP and two prospective non-comparative studies. Friedlander et al. reported that, with a follow-up period of up to 72 months, there was no difference in biochemical recurrence-free survival rates for bladder neck sparing and non-sparing groups after adjusting for pathological stage, grade, baseline PSA and margin status (HR 1.20, 95% CI 0.62–2.31, *p* = 0.596) [9]. BCR was defined, unusually, as PSA greater than 0.1 ng/ml but the number of patients in each group was not clearly reported. Bianco et al. reported that the 7-year estimated disease-free probability was 78% for patients with negative margins versus 54% for those with positive ones (*p* = 0.0001). At a multivariate analysis, Gleason score, PSA, pathological stage and positive margins were predictive for cancer recurrence. [36] In this study, however, the cut-off definition for BCR was not provided. Licht et al. reported that the incidence of BCR was similar in both groups, BNP and non-BNP. In non-BNP group, 64 of 73 patients (88%) were free of disease with 8 (11%) PSA-only or local failures compared with 87 of 98 (89%) disease-free and 9 (9.2%) PSA or local failures in the BNP group [30]. In this study, the cut-off definition for BCR was PSA greater than 0.6 ng/ml and evidence of local recurrence was obtained by a positive vesicourethral anastomotic biopsy performed under transrectal ultrasound guidance. Golabek et al. reported that PSA levels greater than 0.20 ng/ml were found in 14 patients (7.1%), six cases in patients with negative surgical margins (2.8%) and eight in patients with positive SM (9.3%). Surgical margin status showed a significant effect on the 3-year biochemical recurrence-free survival, with a higher percentage of men without PSA recurrence among those with negative surgical margins (89.9 versus 55.8%, respectively; *p* < 0.001) [11]. Given the conflicting reports and the limited number of studies comparing BCR between BNP and non-BNP with often different or not clear cut-off reported, a definitive evaluation cannot be made.

## Conclusion

Bladder neck preservation technique has been extensively adopted during the past decades in open, laparoscopic and robot-assisted radical prostatectomy. Evidence reported in this review suggests that this procedure may increase base-positive margins. Further studies are needed to better investigate the impact of this technique on oncological outcomes. A future paradigm could include modification of intended approach to bladder neck dissection when anterior base lesions are identified on pre-operative MRI or sector template biopsies.
